# Quantitation of Cirtuvivint (SM08502) in Human Plasma by LC–MS/MS

**DOI:** 10.1002/bmc.70565

**Published:** 2026-08-18

**Authors:** Julianne L. Holleran, Joshua J. Deppas, Ye Feng, Michael J. Hochman, Evan C. Chen, Maximilian Stahl, Steven D. Gore, Timothy W. Synold, Jan H. Beumer, Raman Venkataramanan, Robert A. Parise

**Affiliations:** ^1^ Cancer Therapeutics Program UPMC Hillman Cancer Center Pittsburgh Pennsylvania USA; ^2^ Department of Pharmaceutical Sciences, School of Pharmacy University of Pittsburgh Pittsburgh Pennsylvania USA; ^3^ Department of Medical Oncology and Therapeutics Research City of Hope Comprehensive Cancer Center Duarte California USA; ^4^ Winship Cancer Institute Emory University Atlanta Georgia USA; ^5^ Department of Medical Oncology Dana‐Farber Cancer Institute Boston Massachusetts USA; ^6^ Department of Internal Medicine, Section of Medical Oncology and Hematology Yale University School of Medicine and Yale Comprehensive Cancer Center New Haven Connecticut USA; ^7^ Division of Cancer Treatment and Diagnosis, Investigational Drug Branch, Cancer Therapy Evaluation Program National Cancer Institute Bethesda Maryland USA; ^8^ Department of Oncology and Division of Clinical Pharmacology Johns Hopkins University School of Medicine Baltimore Maryland USA; ^9^ Department of Pathology, School of Medicine University of Pittsburgh Pittsburgh Pennsylvania USA

**Keywords:** cirtuvivint, human plasma, LC–MS/MS, pharmacokinetics, SM08505, validation

## Abstract

CDC‐like kinases (CLK) and dual‐specificity tyrosine‐regulated kinases (DYRK) are protein kinases involved in various cellular functions, mRNA splicing, and DNA damage repair. CLK/DYRK kinases have been implicated in many disorders such as diabetes, neurodegenerative diseases, and cancer. Cirtuvivint (SM08502) is an orally bioavailable, first‐in‐class pan‐CLK and pan‐DYRK inhibitor that modulates pre‐mRNA splicing and has shown the ability to inhibit cancer cell growth in vitro and reduce tumor burden in vivo. This has led to the administration of cirtuvivint in phase I clinical trials. To quantitate cirtuvivint, we have developed and validated an LC–MS/MS method in human plasma. The assay is simple and robust and consists of a protein precipitation, dilute‐and‐shoot extraction method using 20 μL of plasma, chromatographic separation with a Phenomenex Kinetex C18, and a gradient mobile phase system consisting of 0.1% formic acid in water and acetonitrile. The chromatographic method is followed by mass spectrometric detection with a SCIEX 4500 tandem mass spectrometer. The method has a 5‐min run time and is linear from 5 to 1000 ng/mL. The assay met the criteria outlined by the US Food and Drug Administration guidance for bioanalytical method validation and will support ongoing and future clinical studies defining cirtuvivint pharmacokinetics.

## 1 Introduction

Dysregulation of pre‐mRNA splicing is increasingly recognized as an important feature of cancer biology and has been implicated in tumor development and progression across multiple malignancies. Alternative splicing can generate aberrant transcript isoforms that disrupt cellular regulatory pathways and promote oncogenic signaling. Central to the regulation of alternative splicing are CDC‐like kinases (CLK1‐CLK4) and dual‐specificity tyrosine‐regulated kinases (DYRK), which phosphorylate serine/arginine‐rich splicing factors and thereby modulate spliceosome assembly, exon recognition, and transcript selection. Aberrant CLK and DYRK signaling have been observed in multiple malignancies and have been associated with widespread alterations in splicing programs associated with tumor cell survival (Song et al. 2023; Van Goubergen et al. 2025).

Pharmacologic inhibition of CLK and DYRK kinases has been shown to disrupt phosphorylation of splicing factors, leading to altered splice‐site selection and broad transcriptome remodeling (Corr et al. 2023; Song et al. 2023). Preclinical studies of CLK and DYRK inhibition demonstrate downstream effects on genes associated with cell‐cycle control, DNA damage response (DDR), and oncogenic signaling networks (Corr et al. 2023). Splicing‐dependent modulation of the Wnt signaling pathway has also been identified as an important consequence of CLK inhibition, as mis‐splicing of Wnt pathway components can attenuate beta‐catenin–mediated transcription, and cause reduced tumor cell proliferation in preclinical models (Tam et al. 2020). Together, these findings support targeting splicing regulation as a therapeutic strategy in cancer.

Cirtuvivint (SM08502) is an orally bioavailable, first‐in‐class small‐molecule inhibitor of pan‐CLK and pan‐DYRK kinases. Preclinical investigations have shown that cirtuvivint reduces phosphorylation of splicing factors, resulting in widespread changes in alternative splicing and downstream modulation of oncogenic transcriptional programs (Corr et al. 2023; Tam et al. 2020). These splicing‐dependent effects have been associated with suppression of cancer‐relevant signaling pathways, including Wnt signaling, providing a mechanistic rationale for continued clinical evaluation of cirtuvivint as a splicing‐modulating therapeutic agent (Corr et al. 2023; Tam et al. 2020). Recent transcriptomic analyses of pediatric central nervous system (CNS) tumors have further highlighted the oncogenic relevance of splicing dysregulation and identified CDC‐like kinase 1 (CLK1) as a potential splicing dependency. In these studies, aberrant inclusion of an active CLK1 isoform was associated with poor clinical outcomes, and pharmacologic inhibition of CLK1 with cirtuvivint disrupted cancer‐relevant splicing programs and suppressed tumor cell proliferation in preclinical models (Naqvi et al. 2025). Cirtuvivint is currently under investigation in four Phase I and II clinical trials as a monotherapy and in combination with DNA‐targeting anticancer agents [clincaltrials.gov, date accessed: 01/29/2026]. Despite increasing mechanistic understanding of cirtuvivint's biological activity, there is not a detailed pharmacokinetic analytical method described in the literature. Accurate measurement of circulating cirtuvivint concentrations is essential to support dose optimization and pharmacokinetic–pharmacodynamic analyses in clinical studies.

Liquid chromatography–tandem mass spectrometry (LC–MS/MS) is widely used for the quantitative bioanalysis of small‐molecule therapeutics because of its high sensitivity, selectivity, and robustness. To date, no validated LC–MS/MS method for the quantitation of cirtuvivint in human plasma has been described, preventing rigorous pharmacokinetic characterization in ongoing and future clinical trials.

The objective of the present study was to develop and validate a sensitive, selective, and reproducible LC–MS/MS method for the quantitation of cirtuvivint in human plasma in accordance with the guidelines outlined by the US Food and Drug Administration (US FDA). This assay is intended to support pharmacokinetic evaluation of cirtuvivint in upcoming cancer clinical trials and to provide an analytical foundation for future exposure–response and metabolism studies.

### 1.1 Chemicals and Reagents

Cirtuvivint (99.9% purity) was obtained from Biosplice Therapeutics (San Diego, CA, United States), while erlotinib (HCl salt, internal standard (IS), > 99% purity) was purchased from LC Laboratories (Woburn, MA, United States). Acetonitrile and water (all LC–MS grade) were purchased from Honeywell Burdick & Jackson (Muskegon, MI, United States). LC–MS grade formic acid was purchased from CovaChem (Loves Park, IL, United States). Control EDTA human plasma was purchased from Lampire Biological Laboratories (Pipersville, PA, United States). Nitrogen for mass spectrometric applications was purified with a nitrogen generator (Parker Balston, Haverhill, MA, United States).

### 1.2 Chromatography

The LC system consisted of a SCIEX (Framingham, MA, United States) Exion LC 30 AC autosampler and 2 Exion LC AC pumps flowing into a Phenomenex (Torrance, CA, United States) Kinetex C18 (2.6 μm, 50 × 2.1 mm, 100 Å) column using a gradient elution program. The temperature of the autosampler was set at 15°C, while the column oven was kept at 30°C. Needle wash solution consisted of acetonitrile:water:isopropanol:methanol (25:25:25:25, *v/v/v/v*). Mobile phase solvent A consisted of 0.1% formic acid in water (*v/v*) and mobile phase solvent B consisted of 0.1% formic acid in acetonitrile (*v/v*). The initial mobile phase composition was 85% solvent A and 15% solvent B. From 0 to 2.5 min, at a 0.4 mL/min flow rate, solvent B was increased to 35%; then, at 2.6 min, solvent B was increased to 80% and maintained until 4.0 min, while flow rate was also increased to 0.8 mL/min. Between 4.0 and 4.1 min, solvent B was returned to the initial composition with a flow rate of 0.8 mL/min, and this was maintained until the end of the run. Between 0–0.8 min and 3.0–6 min, the LC flow was diverted to waste.

### 1.3 Mass Spectrometry

Detection was accomplished with a SCIEX 4500 tandem mass spectrometer (Framingham, MA, United States) utilizing electrospray ionization in positive‐ion multiple reaction monitoring (MRM) mode. The settings of the mass spectrometer in positive mode scanning parameters were as follows: curtain gas 20, collision gas 9, ion spray voltage 5000 V, probe temperature 500°C, GS1 40, GS2 40, DP of 20 V, EP of 10 V, CXP of 6 V, and the ce was set at 40 V. The MRM *m/z* transitions monitored were as follows: 428.3 > 371.0 (quantifier) and 428.3 > 343.0 (qualifier) for cirtuvivint (Figure S1), 394.0 > 278.0 for erlotinib (Figure S2). The dwell time was set at 50 ms for all transitions. Control of the LC system and mass spectrometer as well as data collection was accomplished with Analyst software (Version 1.7.2).

### 1.4 Preparation of Calibration Standards and Quality Control (QC) Samples

Stock solutions of cirtuvivint (1 mg/mL) were prepared by dissolving in DMSO, while stock solutions of erlotinib (1 mg/mL) were prepared by dissolving in methanol. Cirtuvivint stock solution was sequentially diluted in DMSO to obtain 0.1, 0.01, 0.001, and 0.0001 mg/mL working stock solutions for calibrators. These solutions were spiked in human plasma to produce the following cirtuvivint concentrations: 5, 10, 50, 100, 200, 500, and 1000 ng/mL. For each calibration series, zero and blank samples were also prepared from control plasma. QC stock solutions were prepared independently and diluted in DMSO to obtain 0.1, 0.01, 0.001, and 0.0001 mg/mL working stock solutions for QCs. These solutions were diluted in human plasma to produce the following QC samples: lower limit of quantification (LLOQ) 5 ng/mL, QC Low (QCL) 15 ng/mL, QC Mid (QCM) 150 ng/mL, and QC High (QCH) 750 ng/mL. In addition, erlotinib stock solution was diluted to 500 ng/mL in acetonitrile as an IS working stock. All the stock and working solutions were stored at −80°C.

### 1.5 Sample Preparation

A volume of 20 μL of the calibrator, QC, or unknown plasma sample was pipetted into a microfuge tube and 10 μL of 500 ng/mL erlotinib was added. A total of 100 μL of acetonitrile was then added, followed by vortexing for 2 min on a Vortex Genie‐2 set at 10 (Model G‐560 Scientific Industries, Bohemia, NY, United States). Samples were centrifuged at 17,000 ×*g* at 20°C for 10 min. A volume of 50 μL of supernatant was transferred to autosampler glass vials containing 50 μL of water; samples were briefly vortexed, followed by injection of 3 μL into the LC–MS/MS system.

### 1.6 Validation Procedures

#### 1.6.1 Calibration Curve and LLOQ

Calibration standards and blanks were prepared in human EDTA plasma and analyzed in triplicate, and in three independent runs, to establish the calibration range with acceptable accuracy and precision. Standard curves were constructed individually by plotting the analyte‐to‐IS area ratio versus the nominal concentrations in each sample. Standard curves were fitted by linear regression with weighting by 1/y^2^, followed by back‐calculation of concentrations. The deviations of these back‐calculated concentrations from the nominal concentrations were expressed as a percentage of the nominal concentration.

#### 1.6.2 Accuracy and Precision

The accuracy and precision of the assay were determined by analyzing samples at the LLOQ, QCL, QCM, and QCH concentrations (six replicates each, in three independent runs), together with independently prepared triplicate calibration curves. Accuracy was calculated at each test concentration as follows: (mean measured concentration/nominal concentration) × 100%.

Assay precision was calculated by ANOVA as previously described (Rosing et al. 2000), by using SPSS 27.0 for Windows (SPSS Inc., Chicago, IL, United States). Back‐calculated concentrations of calibration and QC samples were entered with the run number as factor. From the resulting mean squares of the within runs and mean squares of the between runs, the intra‐assay and inter‐assay precisions were calculated.

#### 1.6.3 Selectivity and Specificity

To investigate whether endogenous matrix constituents interfered with the assay, six individual batches of control, drug‐free human EDTA plasma were processed and analyzed according to the described procedure. Responses of the analyte at the LLOQ concentration were compared with the response of the blank samples.

The crosstalk of cirtuvivint into the IS signal, and vice versa, was characterized by monitoring MRM channels of separately spiked samples of 10,000 ng/mL cirtuvivint or 1000 ng/mL of IS and quantitating the response in the other channel.

Carry‐over was assessed by separately injecting plasma samples with 10,000 ng/mL cirtuvivint, followed by serial plasma blank injections.

#### 1.6.4 Extraction Recovery and Matrix Effect

The extraction recovery of cirtuvivint from plasma was determined by comparing the absolute response of an extract of control plasma to which the analyte had been added after protein precipitation, with the absolute response of an extract of plasma to which the same amount had been added before protein precipitation. The matrix effect by plasma matrix components was defined as the change of the absolute response of an extract of control plasma to which the analyte had been added after the protein precipitation relative to the absolute response of solvent to which the same amount of the analyte had been added. Experiments were performed in replicates of four at the QCL, QCM, and QCH concentrations.

#### 1.6.5 Stability

Stock solution stabilities were determined using freshly prepared stock solutions in DMSO and comparing them to stock solutions left on the benchtop at room temperature (RT) for 6 h (benchtop stability) or stored at −80°C for 8 months (long‐term stability). Stock solutions were diluted to 1000 ng/mL (benchtop stability) or 100 ng/mL (long‐term stability) in control plasma and prepared in replicates of three. Concentrations used to assess stability were derived by the same calibration curve and QC series.

The stability of cirtuvivint in human EDTA plasma at −80°C was determined by assaying samples before and after storage for 7 months. The effect of three freeze/thaw cycles on analyte concentrations in plasma was evaluated by assaying samples after they had been frozen (−80°C) and thawed on three separate days and comparing the results with those of freshly prepared samples. The stability of cirtuvivint in plasma during sample preparation was evaluated by assaying samples before and after 4 h of storage at room temperature and ambient light. To evaluate the stability of cirtuvivint in extract, six QCL samples were processed, pooled, and injected into the LC–MS/MS system every hour for 72 h. To evaluate the reinjection reproducibility of cirtuvivint extract samples in the autosampler, QC samples and calibration curves were re‐injected approximately 72 h after the first injection: concentrations derived from the second injection were compared with those derived from the first injection using the initial duplicate calibration curve (U.S. Department of Health and Human Services Food and Drug Administration 2022). The results of the second runs were expressed as a percentage of their respective values in the first runs. All stability testing in plasma was performed in replicates of four at the QCL and QCH concentrations.

#### 1.6.6 Additional Validation Items

Hemolyzed plasma conditions were assessed during validation: hemolyzed plasma was obtained by adding 10% (*v/v*) pre‐hemolyzed whole blood to human EDTA plasma samples. Four replicates of blanks, LLOQ, QCL, and QCH samples were employed for this assessment.

Dilutional integrity was tested by preparing plasma samples (*n* = 5) at 10,000 ng/mL and analyzed after 10‐fold dilution (to 1000 ng/mL) with control plasma.

The potential for phospholipids to interfere in analysis was also assessed by monitoring known phospholipid MRM channels in a 750 ng/mL plasma sample (Little et al. 2006; Zhang and Wujcik 2009).

### 1.7 Application of the Assay

To demonstrate the applicability of the assay, we analyzed plasma samples from one patient receiving 60 mg cirtuvivint on a 2‐days‐on/5‐days‐off dosing schedule. The patient had signed informed consent on an institutional review board approved protocol (ClinicalTrials.gov ID NCT06484062). Blood samples were collected up to 24 h after initial dosing. Pharmacokinetic parameters were determined non‐compartmentally with Phoenix WinNonlin software (version 8.4.0.6172).

### 1.8 External Validation

Blinded QCs were prepared at three levels and provided by the Analytical Pharmacology Core at City of Hope Cancer Center (Duarte, CA, United States).

### 1.9 Incurred Sample Reanalysis

To further assess assay reproducibility, incurred sample reanalysis was performed using clinical plasma samples obtained from the pharmacokinetic study.

## 2 Results and Discussion

### 2.1 Validation of the Assay

#### 2.1.1 Chromatography

**FIGURE 1:**
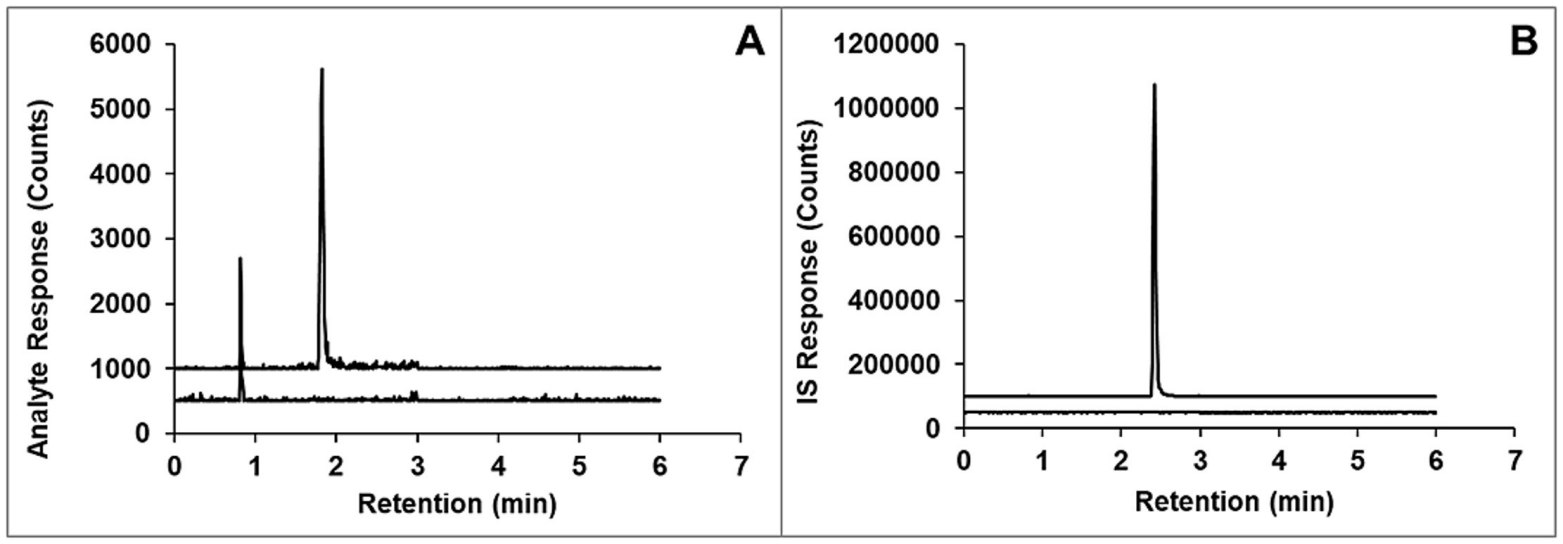
Representative chromatograms of (A) cirtuvivint (*m/z* 428.3 > 371.0; 1.72 min) added to control human plasma at the LLOQ concentration of 5 ng/mL (top trace with an offset of 1 · 10^3^ counts) and control human plasma (bottom trace with an offset of 500 counts); (B) erlotinib (*m/z* 394.0 > 278.0; 2.36 min) added to control human plasma at a concentration of 250 ng/mL (top trace with an offset of 100 · 10^3^ counts) and control human plasma (bottom trace with an offset of 50 · 10^3^ counts).

The retention times of both cirtuvivint and erlotinib were 1.72 and 2.36 min, respectively. This corresponds to a capacity factor of 1.7 for cirtuvivint and 2.4 for erlotinib in conjunction with a void time of 0.3 min. Representative chromatograms of cirtuvivint and erlotinib in human plasma (blank and LLOQ) are displayed in Figure 1A,B.

#### 2.1.2 Calibration Curve and LLOQ

The selected assay range of 5–1000 ng/mL fulfilled the FDA criteria for the LLOQ concentration (sensitivity) and the calibration curve (accuracy within ±15% of nominal concentrations) and precision ≤ 15% (LLOQ within ±20% and ≤ 20%), with 75% and a minimum of six non‐zero calibrators meeting these criteria in each run (U.S. Department of Health and Human Services Food and Drug Administration 2018). Accuracies and precisions for each concentration from triplicate calibration curves prepared on three separate days are reported in Table S1. At most concentrations, the mean square of the within runs was greater than the mean square of the between runs, indicating that there was no significant additional variability due to the performance of the assay in different runs (Rosing et al. 2000). A representative calibration curve and the corresponding correlation and regression coefficient as well as corresponding accuracies are shown in Figure S3A,B.

#### 2.1.3 Accuracy and Precision

The intra‐ and inter‐assay accuracies and precisions for the tested concentrations (LLOQ, QCL, QCM, QCH) were all within the defined acceptance criteria (accuracy: ±15% of nominal concentrations, except ±20% at LLOQ; precision: ≤ 15% CV, except ≤ 20% CV at LLOQ), as summarized in Table 1.

**TABLE 1:** Assay performance data for the quantitation of LLOQ, QCL, QCM, and QCH concentrations of cirtuvivint in human plasma.

Concentration (ng/mL)	Intra‐assay accuracy (%)	Inter‐assay accuracy (%)	Intra‐assay precision (%)	Inter‐assay precision (%)
5 (LLOQ)	86.3–98.6	90.8	12.4	5.4
15 (QCL)	90.9–104.9	98.0	7.3	6.5
150 (QCM)	94.4–101.9	98.5	5.4	3.1
750 (QCH)	93.0–100.4	96.1	5.4	3.3

*Note: n* = 18; six‐fold results, each in three separate runs, for each concentration. The mean square of the within runs was greater than the mean square of the between runs, indicating that there was no significant additional variation due to the performance of the assay in different runs (Rosing et al. 2000).

Abbreviations: LLOQ, lower limit of quantitation; QCH, QC High; QCL, QC Low; QCM, QC Mid.

#### 2.1.4 Selectivity and Specificity

Chromatograms of six individual control plasma samples contained no co‐eluting peaks > 20% of the analyte areas at the LLOQ concentration (interference ≤ 0.43%) and no co‐eluting peaks > 5% of the IS area (interference ≤ 0.01%).

The mean crosstalk of the 1000 ng/mL IS into the cirtuvivint MRM channel was 0.04%. Scaling this to the assay IS concentration of 250 ng/mL corresponds to 0.1 ng/mL cirtuvivint (2% of the LLOQ). About 10,000 ng/mL cirtuvivint produced 0.001% as mean crosstalk into the IS MRM channel that would correspond to 0.05 ng/mL at the 5000 ng/mL ULOQ (0.02% of the applied IS concentration).

Carryover in the developed assay was lower than 0.015% of the original 10,000 ng/mL sample signal in sequential blank plasma injections (Figure S4), which corresponded to 2.5% of the LLOQ area. Defining the maximum carryover as 20% of the LLOQ area, this would mean that only samples with a concentration of more than approximately 80,000 ng/mL could result in unacceptable carryover into the next sample.

#### 2.1.5 Extraction Recovery and Matrix Effect

The recoveries of cirtuvivint ranged from 101% to 111% (CV 6.6%–17.0%), while matrix effect ranged from 0.7% to 10.5% (CV 7.8%–12.4%), as reported in Table 2. Although the extraction and matrix effect displayed some variability, this did not affect the performance of the assay, as is evident from the appropriate accuracies and precisions across the concentration range (Table 1 and Table S1).

**TABLE 2:** Recoveries of cirtuvivint from human plasma and respective matrix effects in human plasma extract, with coefficients of variation (CV).

Concentration (ng/mL)	Recovery (%)	CV (%)	Matrix effect (%)	CV (%)
15 (QCL)	104.2	8.3	0.7	6.6
150 (QCM)	111.0	15.6	6.9	17.0
750 (QCH)	101.0	10.1	10.5	16.7

*Note: n* = 4, for each concentration.

Abbreviations: QCH, QC High; QCL, QC Low; QCM, QC Mid.

#### 2.1.6 Stability

Stock solution stability of cirtuvivint in DMSO after 6 h at RT was 97.4% (4.7% CV) using replicates of 3, and after 8 months at −80°C was 100.4% (5.4% CV), using replicates of 5.

Stability data for cirtuvivint in human plasma are summarized in Table 3. The stability after three freeze–thaw cycles (−80°C to RT) was between 99.5% and 100.6% (CV ≤ 11.3%). Benchtop stability in plasma after 4 h at RT with ambient light ranged between 92.7% and 101.8% (CV ≤ 5.7%). Long‐term stability in plasma after 8 months at −80°C was adequate with recovery between 102.9% and 110.9% (CV ≤ 8.2%). Accuracy of the back‐calculated concentrations of plasma extracts of cirtuvivint at QC levels, when reconstituted and kept in the autosampler for 72 h, ranged from 100.3% to 101.1% (CV ≤ 6.9%).

**TABLE 3:** Stability of cirtuvivint in human plasma under varying conditions.

Storage condition	Concentration (ng/mL)	Stability (%)	CV (%)
4 h room temperature	15 (QCL)	101.8	5.7
	750 (QCH)	101.8	3.3
3 freeze–thaw cycles −80°C	15 (QCL)	99.5	11.3
	750 (QCH)	100.6	4.6
72 h reinjection reproducibility	15 (QCL)	100.3	6.9
	150 (QCM)	99.0	2.4
	750 (QCH)	101.1	2.9
8 months at −80°C	15 (QCL)	102.9	8.2
	750 (QCH)	110.9	6.1

*Note:* Stability experiments were performed in replicates of *n* = 4.

Abbreviations: QCH, QC High; QCL, QC Low; QCM, QC Mid.

#### 2.1.7 Additional Validation Items

Hemolyzed plasma samples did not result in extra peaks in blank samples, and quantitation was not affected with an accuracy of 106.3% (CV of 9.3%) compared with non‐hemolyzed samples.

Dilutional integrity of the assay was confirmed with 95.1% accuracy (CV of 6.0%). Endogenous phospholipids did not co‐elute with cirtuvivint MRM channel, limiting the risk of interference (Figure S5).

In case extra assurance of identity is desired by means of a qualifier ion, MRM *m/z* transition 321.2 > 235.2 can be monitored. This transition has an intensity of ~68% of the quantifier transition *m/z* 428.3 > 371.0, with a suggested relative tolerance of ±50%, which translates to 34%–102% (*Commission Decision (2002/657/EC) of 12 August 2002 Implementing Council Directive 96/23/EC Concerning the Performance of Analytical Methods and the Interpretation of Results* 2002).

### 2.2 External Validation

Blinded QCs (three levels) prepared by an external laboratory were analyzed in replicates of 5. Upon analysis and unblinding, the median difference measured as bias was 10% (range −2% to 12%).

### 2.3 Incurred Sample Reanalysis

Seven patient samples spanning the concentration–time profile were reanalyzed after storage at −80°C for 204 days. Reanalyzed concentrations differed from the original values by −4.4% to +6.2%, with all samples meeting the ±20% acceptance criterion.

### 2.4 Development

Method development began with MS tuning to optimize the parameters for both cirtuvivint and erlotinib (IS) to obtain sensitive and specific detection. Cirtuvivint and IS were infused separately into the MS with a mobile phase containing water:acetonitrile 1:1, both with 0.1% formic acid (*v/v*). The mass spectrometer was first operated in ESI positive mode, and the source parameters were tuned to maximize the signal intensities of the precursor ions. Positive and negative ionization modes were tested, and positive ion mode produced better ionization, and scans revealed high intensity *m/z* 428.3 and 394.0 singly protonated ions for cirtuvivint and IS, respectively. Product ion scans for cirtuvivint revealed a predominant product ion trace of *m/z* 371.0, which was chosen as the quantifier ion, and a lesser product ion of 343.0, which was chosen as the qualifier ion. For the internal standard, the predominant product ion of 278.0 was obtained and selected for quantification.

The LC method was explored by injection of neat standards into the LC–MS/MS system, evaluating three different columns: Phenomenex Kinetics C18 (2.6 μm, 50 × 2.1 mm, 100 Å), Phenomenex Luna Phenyl‐Hexyl (3 μm, 50 × 2.0 mm, 100 Å), and Restek Raptor Biphenyl (2.7 μm, 50 × 2.1 mm, 90 Å). All the three columns were tested using the same scouting gradient program, with 0.1% formic acid in water as solvent A (*v/v*) and 0.1% formic acid in acetonitrile as solvent B (*v/v*): from 0 to 6 min the solvent B was increased from 10% to 80%, followed by re‐equilibration for 2 min. In these tests, the flow rate was set at 0.4 mL/min and the column oven at 40°C. Although all three columns resulted in similar retention and peak shape, the Kinetex C18 column showed slightly less tailing and the system backpressure was lower, resulting in its selection for further method optimization.

Since an isotopic internal standard was not commercially available, we tested four different potential standards (abiraterone, erlotinib, palbociclib, and venetoclax) using the same HPLC gradient mentioned above. Erlotinib was chosen as the IS because it eluted shortly after cirtuvivint, which reduces the chance of a potential interference with any metabolites that might be generated with therapeutic administration or during future in vitro metabolic studies with cirtuvivint.

The gradient program was then optimized, shortening the run time to 6 min while maintaining a capacity factor of 1.7. Finally, the MRM dwell times were set at 100 ms for all MRM channels, which ensured at least 20 points defined the peak at the LLOQ.

Sample preparation was designed to support high‐throughput analysis of clinical pharmacokinetic samples while maintaining adequate sensitivity, reproducibility, and assay robustness. Protein precipitation was selected as the extraction technique because preliminary studies demonstrated acceptable recovery, low matrix effects, and satisfactory assay accuracy and precision using a simple acetonitrile‐based extraction. More complex extraction approaches, such as liquid–liquid extraction, were not pursued because the performance achieved with protein precipitation met all validation acceptance criteria while providing a rapid and efficient workflow suitable for large clinical studies. Protein precipitation was performed using acetonitrile at a plasma‐to‐solvent ratio of 1:5 (v/v). Acetonitrile was selected because it efficiently precipitated plasma proteins while maintaining high analyte recovery and producing clean chromatograms with minimal matrix interference. The organic extract was initially injected directly into the LC–MS/MS system; however, substantial peak fronting was observed for both cirtuvivint and the internal standard. Therefore, the extract was diluted approximately 1:1 with water prior to injection, which produced symmetrical peak shapes, with injection volumes of 3 μL.

Calibration curves were initially evaluated from 5 to 5000 ng/mL. Linearity was maintained up to 1000 ng/mL, with signal suppression observed at higher concentrations. Therefore, the validated calibration range was set to 5–1000 ng/mL, over which the assay demonstrated acceptable linearity, accuracy, and precision for pharmacokinetic analysis.

### 2.5 Application of the Assay

**FIGURE 2:**
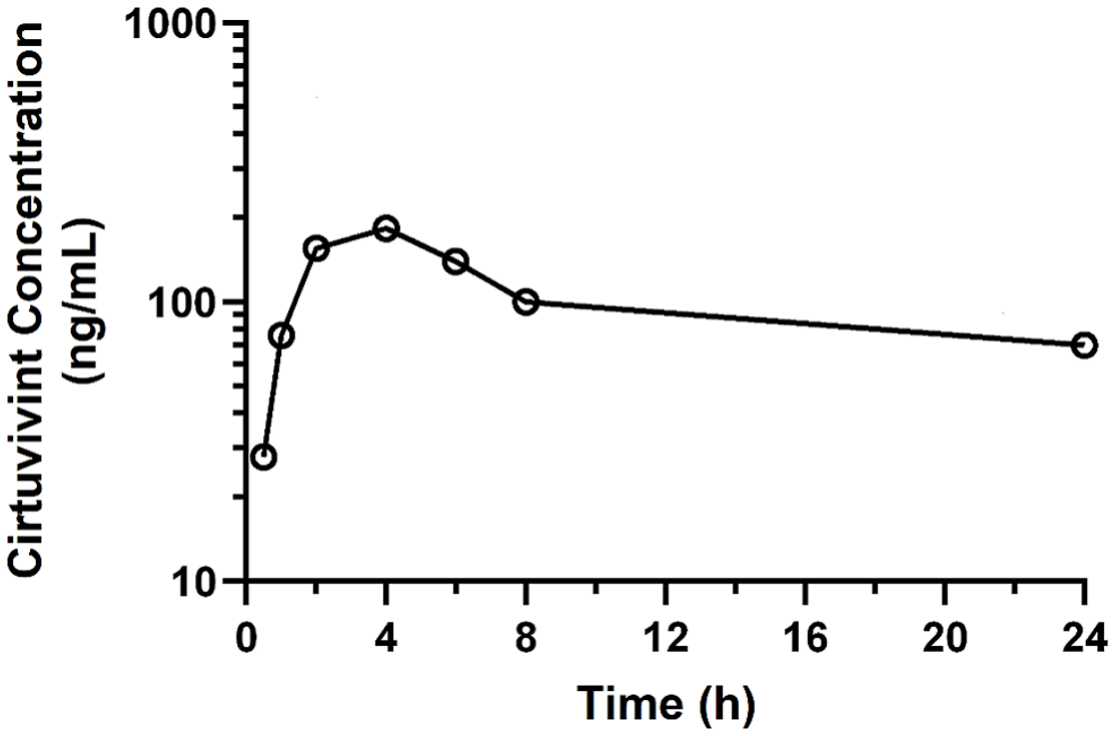
Cirtuvivint (○) plasma concentrations in a patient administered 60 mg of cirtuvivint PO on a 2‐day on and 5‐day off schedule.

All concentrations were within the assay range (Figure 2). After a 60‐mg dose, the observed C_max_ was 184 ng/mL occurring at 4 h post dose and the AUC_0‐∞_ was calculated to be 4.6 μg·h/mL, with an associated apparent clearance of 13.1 L/h and terminal half‐life of 21.5 h. Overall, the assay proved appropriate to accurately quantitate cirtuvivint in human plasma, and it will be a valuable tool in helping to determine the pharmacokinetic parameters and metabolic fate.

## 3 Conclusion

A novel LC–MS/MS method for the quantification of cirtuvivint in human plasma has been developed. It was successfully validated based on the criteria of the US FDA guidance and was suitable to capture clinically relevant concentrations. The assay proved to be accurate, precise, and reliable, with simple and fast sample preparation. This method will offer investigators a robust tool for cirtuvivint quantification and will support PK determination in future clinical trials.

## Funding

Support: Grant R50 CA211241 (NCI). This project used the UPMC Hillman Cancer Center’s Cancer Pharmacokinetics and Pharmacodynamics Facility (CPPF) and was supported in part by award P30 CA47904 and NCI U24CA247643.

## Conflicts of Interest

The authors declare no conflicts of interest.

## Supporting information


**Table S1:** Assay performance data of the calibration samples for cirtuvivint in human plasma.
**Figure S1:** Chemical structure of cirtuvivint (top) and proposed fragmentation pattern of quantifier and qualifier ions (bottom).
**Figure S2:** Chemical structure of erlotinib (top) and proposed fragmentation pattern of product ion (bottom).
**Figure S3:** Representative calibration curve (n = 3 for each concentration) of cirtuvivint in human plasma (response cirtuvivint = 0.00119 · conc + 0.000832; R2 = 0.998). (A) Calibration curve is depicted as response ratio versus nominal concentration; (B) as residuals (%) of the back‐calculated relative to the nominal concentrations versus the log transformed concentration.
**Figure S4:** Analytical carry‐over of cirtuvivint described in serial blank human plasma sample injections following a 10,000 ng/mL spiked plasma sample, represented as the mean percent analyte response relative to the 10,000 ng/mL response (error bars represent ±1 SD).
**Figure S5:** Chromatogram of cirtuvivint at 1000 ng/mL and erlotinib at 250 ng/mL (bottom trace corresponding with right y‐axis) with selected phospholipid MRM channels (left y‐axis). For visual purposes phospholipid MRM channels were offset from baseline response in increments of 25 · 103 response counts and, from top trace, MRM channels were as follows: 184 > 184, 758 > 184, 496 > 184, 784 > 184, 760 > 184, 806 > 184, 704 > 184, 524 > 184, 522 > 184, 394 > 278.

## Data Availability

The data that support the findings of this study are available from the corresponding author upon reasonable request.

## References

[bmc70565-bib-0001] 2002. “Commission Decision (2002/657/EC) of 12 August 2002 Implementing Council Directive 96/23/EC Concerning the Performance of Analytical Methods and the Interpretation of Results.” OJ L 221, 17.8.2002, 8–36. Brussels, Belgium.

[bmc70565-bib-0002] Corr, B. R. , M. R. Moroney , E. Woodruff , et al. 2023. “Combination CDC‐Like Kinase Inhibition (CLK)/Dual‐Specificity Tyrosine‐Regulated Kinase (DYRK) and Taxane Therapy in CTNNB1‐Mutated Endometrial Cancer.” bioRxiv. 10.1101/2023.04.04.535570.

[bmc70565-bib-0003] Little, J. L. , M. F. Wempe , and C. M. Buchanan . 2006. “Liquid Chromatography‐Mass Spectrometry/Mass Spectrometry Method Development for Drug Metabolism Studies: Examining Lipid Matrix Ionization Effects in Plasma.” Journal of Chromatography. B, Analytical Technologies in the Biomedical and Life Sciences 833, no. 2: 219–230. 10.1016/j.jchromb.2006.02.011. 16497566

[bmc70565-bib-0004] Naqvi, A. S. , P. J. Sullivan , R. J. Corbett , et al. 2025. “Characterization of Aberrant Splicing in Pediatric Central Nervous System Tumors Reveals CLK1 as a Candidate Oncogenic Dependency.” bioRxiv. 10.1101/2024.08.03.606419.

[bmc70565-bib-0005] Rosing, H. , W. Y. Man , E. Doyle , A. Bult , and J. H. Beijnen . 2000. “Bioanalytical Liquid Chromatographic Method Validation. A Review of Current Practices and Procedures.” Journal of Liquid Chromatography & Related Technologies 23, no. 3: 329–354. 10.1081/jlc-100101455.

[bmc70565-bib-0006] Song, M. , L. Pang , M. Zhang , Y. Qu , K. V. Laster , and Z. Dong . 2023. “Cdc2‐Like Kinases: Structure, Biological Function, and Therapeutic Targets for Diseases.” Signal Transduction and Targeted Therapy 8, no. 1: 148. 10.1038/s41392-023-01409-4. 37029108 PMC10082069

[bmc70565-bib-0007] Tam, B. Y. , K. Chiu , H. Chung , et al. 2020. “The CLK Inhibitor SM08502 Induces Anti‐Tumor Activity and Reduces Wnt Pathway Gene Expression in Gastrointestinal Cancer Models.” Cancer Letters 473: 186–197. 10.1016/j.canlet.2019.09.009. 31560935

[bmc70565-bib-0008] U.S. Department of Health and Human Services Food and Drug Administration . 2018. Guidance for Industry‐Bioanalytical Method Validation. U.S.Department of Health and Human Services; Food and Drug Administration; Center for Drug Evaluation and Research (CDER); Center for Veterinary Medicine (CVM). www.fda.gov/media/70858/download.

[bmc70565-bib-0009] U.S. Department of Health and Human Services Food and Drug Administration . 2022. Guidance for Industry‐M10 BIOANALYTICAL METHOD VALIDATION AND STUDY SAMPLE ANALYSIS. U.S.Department of Health and Human Services; Food and Drug Administration; Center for Drug Evaluation and Research (CDER).

[bmc70565-bib-0010] Van Goubergen, J. , M. Perina , F. Handle , et al. 2025. “Targeting the CLK2/SRSF9 Splicing Axis in Prostate Cancer Leads to Decreased ARV7 Expression.” Molecular Oncology 19, no. 2: 496–518. 10.1002/1878-0261.13728. 39258426 PMC11792998

[bmc70565-bib-0011] Zhang, G. , and C. E. Wujcik . 2009. “Overcoming Ionization Effects Through Chromatography: A Case Study for the ESI‐LC‐MS/MS Quantitation of a Hydrophobic Therapeutic Agent in Human Serum Using a Stable‐Label Internal Standard.” Journal of Chromatography. B, Analytical Technologies in the Biomedical and Life Sciences 877, no. 22: 2003–2010. 10.1016/j.jchromb.2009.05.031. 19520622

