# Supplementary material for: Quantitation of Cirtuvivint (SM08502) in Human Plasma by LC–MS/MS
Source: Biomed Chromatogr. 2026 Aug 18;40(10):e70565. doi: 10.1002/bmc.70565 (PMC13482837; doi:10.1002/bmc.70565)
Supplement: Supplementary file 1 [file BMC-40-e70565-s001.pdf]

Supplementary File

Quantitation of Cirtuvivint (SM08502) in Human Plasma by LC-MS/MS

Julianne L Holleran[1], Joshua J. Deppas[1,2], Ye Feng[3], Michael J. Hochman[4], Evan C. Chen[5], Maximilian Stahl[6], Steven D. Gore[7], Timothy W. Synold[3], Jan H. Beumer[2,8], Raman Venkataramanan[1,2,9], Robert A. Parise[1]

[1]Cancer Therapeutics Program, UPMC Hillman Cancer Center, Pittsburgh, PA
[2]Department of Pharmaceutical Sciences, School of Pharmacy, University of Pittsburgh, Pittsburgh, PA
[3]Department of Medical Oncology and Therapeutics Research, City of Hope Comprehensive Cancer Center, Duarte, CA, United States.
[4]Winship Cancer Institute, Emory University, Atlanta, GA
[5]Department of Medical Oncology, Dana-Farber Cancer Institute, Boston, MA.
[6]Department of Internal Medicine, Section of Medical Oncology and Hematology, Yale University School of Medicine and Yale Comprehensive Cancer Center, New Haven, CT.
[7]Division of Cancer Treatment and Diagnosis, Investigational Drug Branch, Cancer Therapy Evaluation Program, National Cancer Institute, Bethesda, Maryland, USA.
[8] Department of Oncology and Division of Clinical pharmacology, Johns Hopkins University School of Medicine, Baltimore, MD, USA.
[9]Department of Pathology, School of Medicine, University of Pittsburgh, Pittsburgh, PA

Keywords: Cirtuvivint, SM08505, human plasma, LC-MS/MS, validation, pharmacokinetics.

Emails: holljl@upmc.edu; jod102@pitt.edu; parisera@upmc.edu; TSynold@coh.org; yefeng@coh.org; mjhochm@emory.edu; Evan_Chen@DFCI.HARVARD.EDU; maximilian.stahl@yale.edu; steven.gore@nih.gov; beumerj@gmail.com; rv@pitt.edu

Supplemental Table

Suppl.Table 1. Assay performance data of the calibration samples for cirtuvivint in human plasma.

Conc. (ng/mL)	Intra-assay Accuracy (%)	Inter-assay Accuracy (%)	Intra-assay precision (%)	Inter-assay precision (%)
5	96.1-97.4	96.8	8.0	*
10	108.7-112.7	110.6	8.1	*
50	95.0-102.0	98.9	4.1	2.7
100	101.8-112.0	108.1	4.9	4.2
200	103.0-107.0	104.6	6.3	*
500	88.0-97.7	92.4	5.2	4.4
1000	94.6-100.4	98.4	5.7	0.78

n=9; triplicate results, each in 3 separate runs, for each concentration. *The mean square of the within runs was greater than the mean square of the between runs, indicating that there was no significant additional variation due to the performance of the assay in different runs (Rosing et al., 2000).

Supplemental Figures

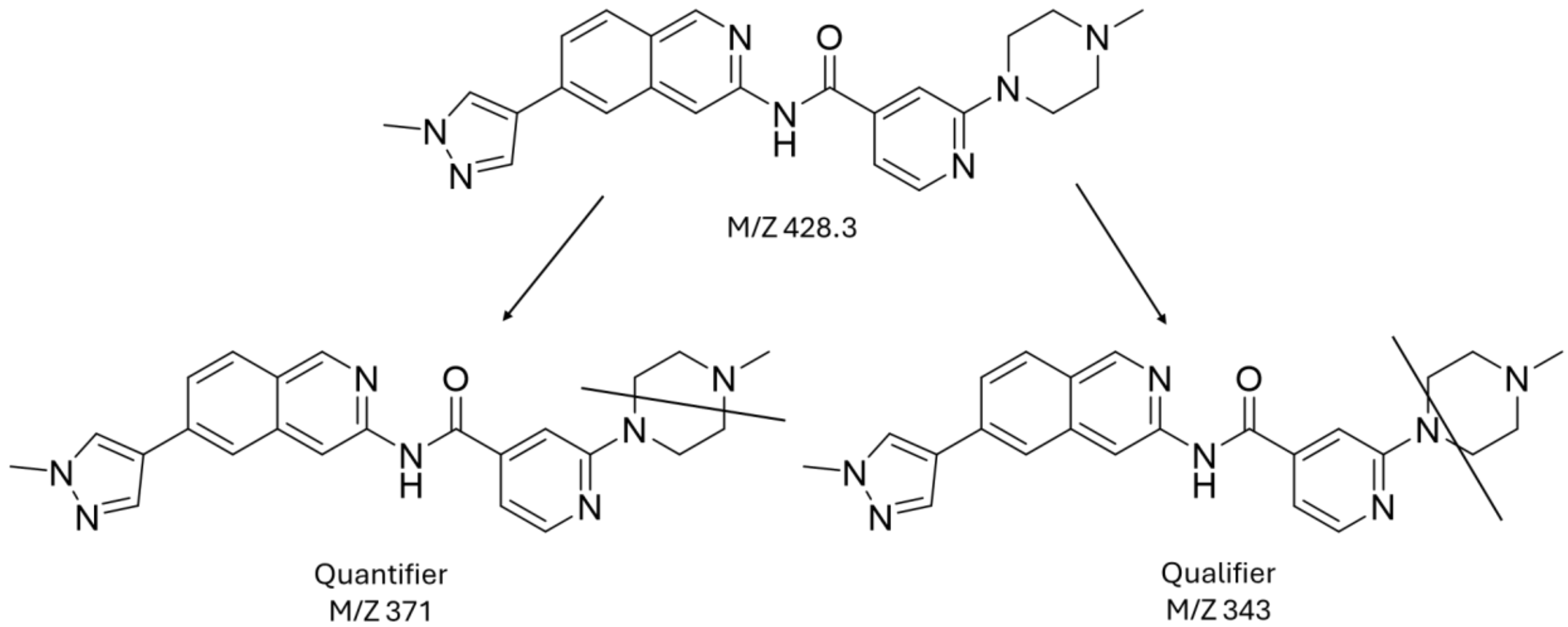

Suppl.Fig. 1. Chemical structure of cirtuvivint (top) and proposed fragmentation pattern of quantifier and qualifier ions (bottom).

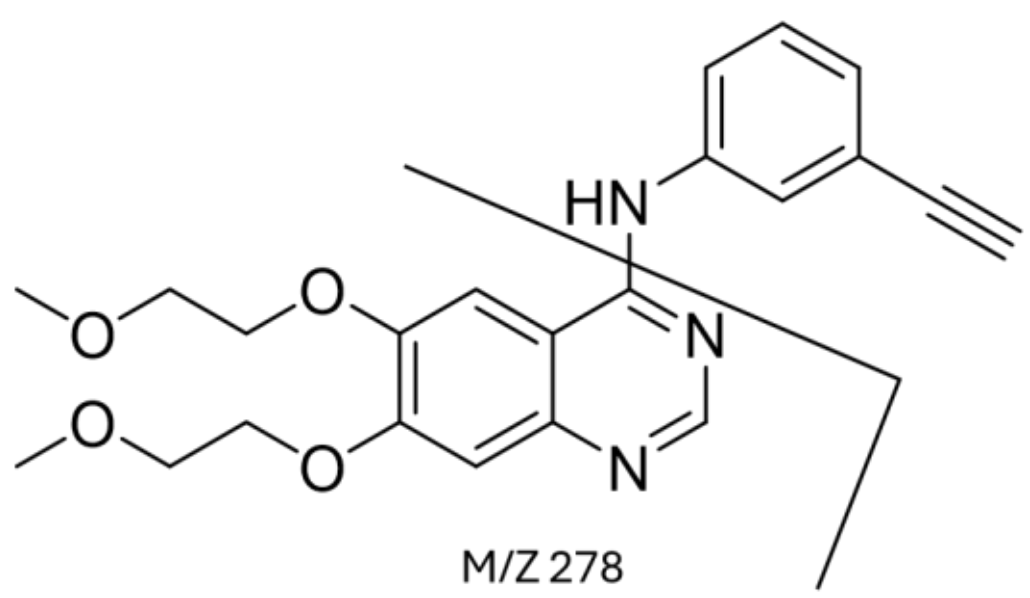

Suppl.Fig. 2. Chemical structure of erlotinib (top) and proposed fragmentation pattern of product ion (bottom).

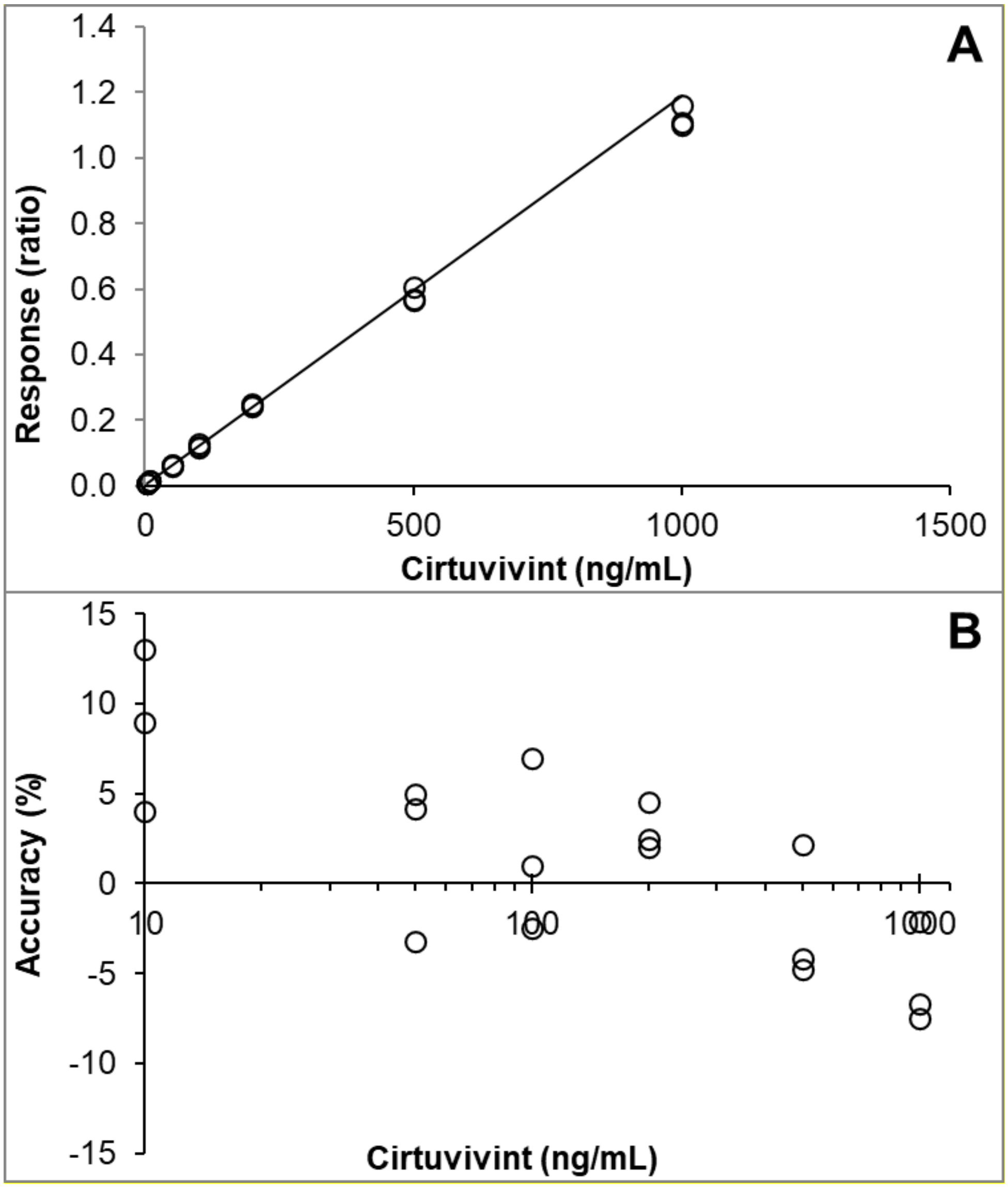

Suppl.Fig. 3. Representative calibration curve (n=3 for each concentration) of cirtuvivint in human plasma (response cirtuvivint = 0.00119•conc + 0.000832; R^2=0.998). A) Calibration curve is depicted as response ratio versus nominal concentration; B) as residuals (%) of the back-calculated relative to the nominal concentrations versus the log transformed concentration.

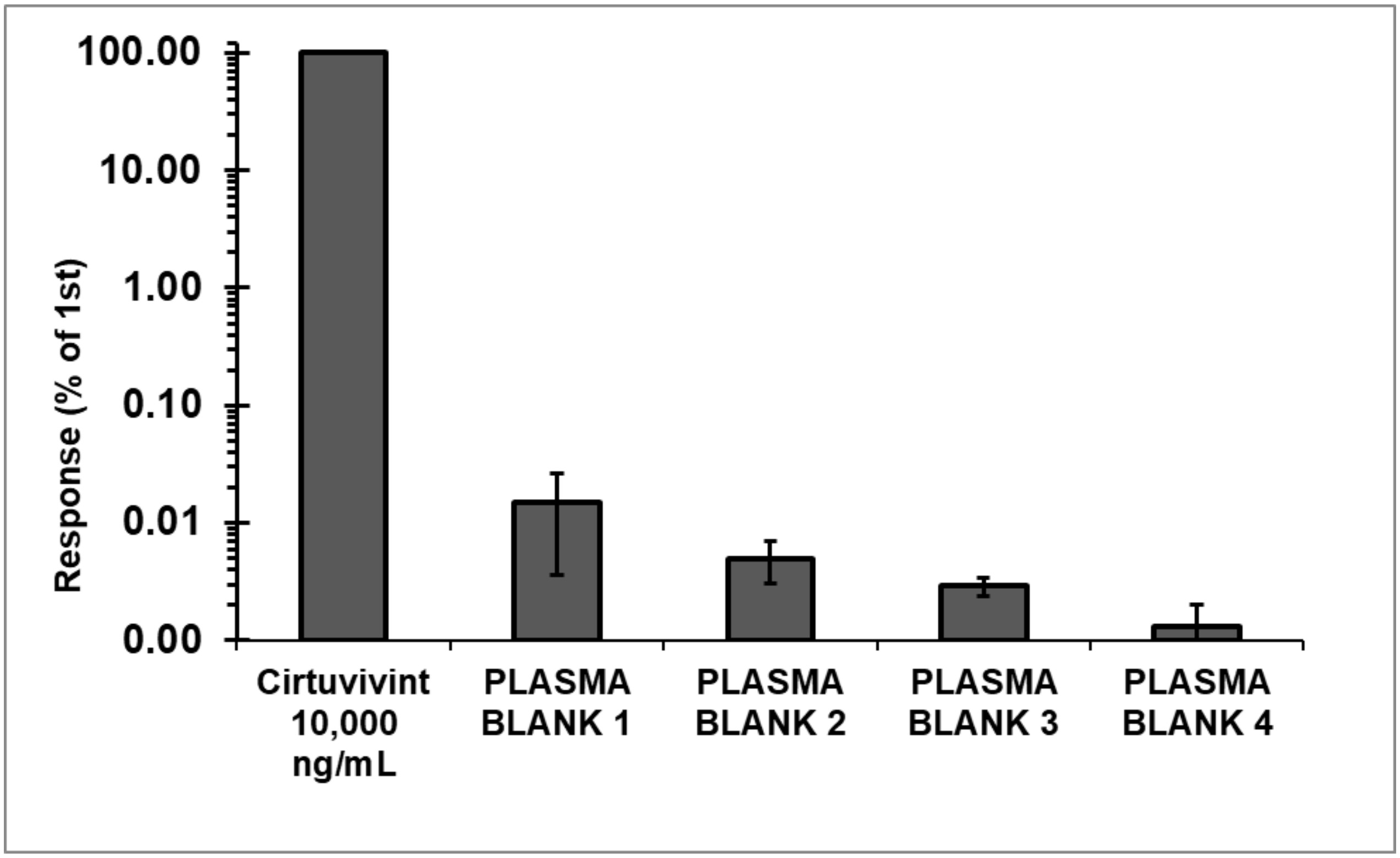

Suppl.Fig. 4. Analytical carry-over of cirtuvivint described in serial blank human plasma sample injections following a 10,000 ng/mL spiked plasma sample, represented as the mean percent analyte response relative to the 10,000 ng/mL response (error bars represent ± 1 SD).

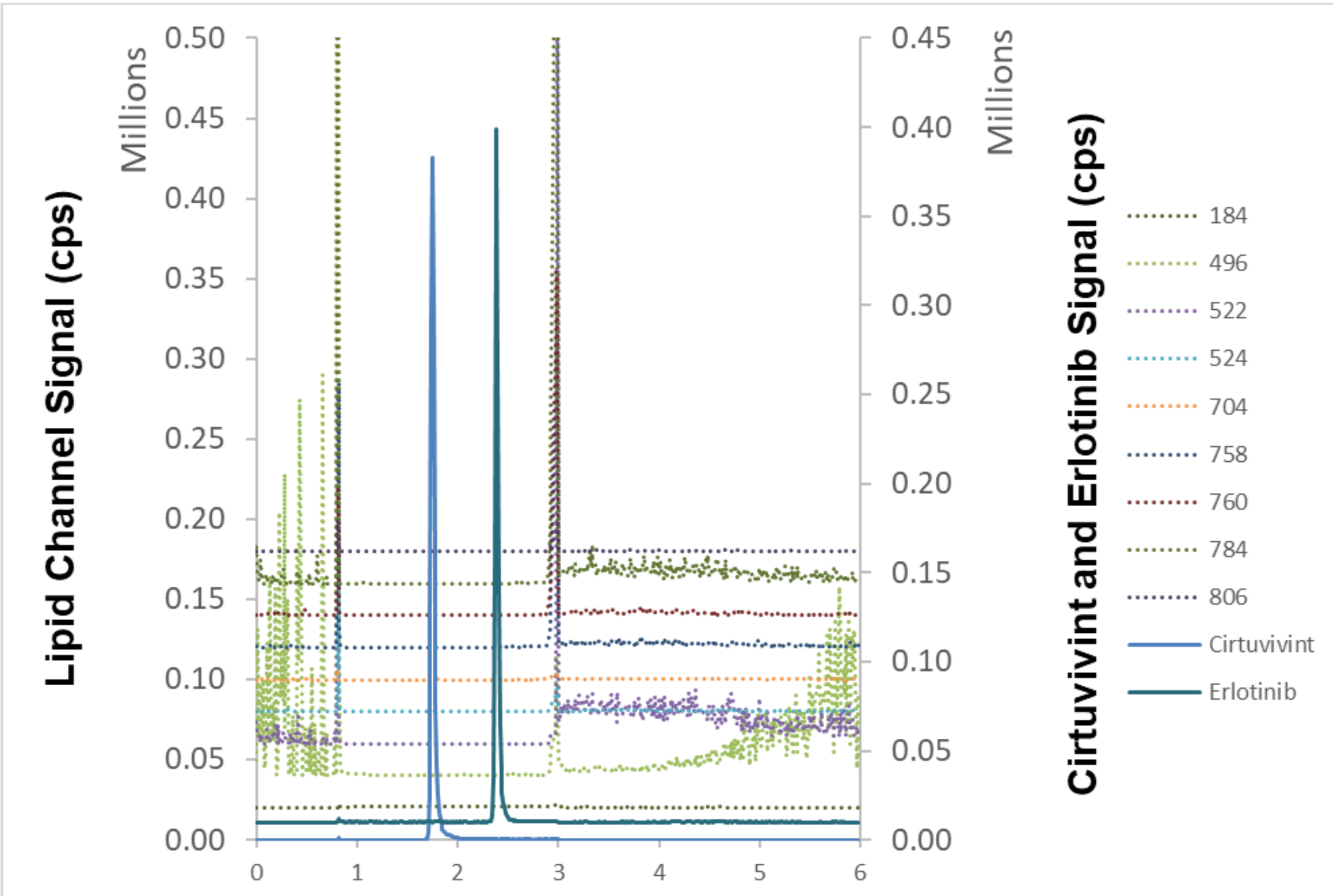

Suppl.Fig. 5. Chromatogram of cirtuvivint at 1000 ng/mL and erlotinib at 250 ng/mL (bottom trace corresponding with right y-axis) with selected phospholipid MRM channels (left y-axis). For visual purposes phospholipid MRM channels were offset from baseline response in increments of $25 \cdot 10^3$ response counts and, from top trace, MRM channels were as follows: 184>184, 758>184, 496>184, 784>184, 760>184, 806>184, 704>184, 524>184, 522>184, 394>278.
